# Analysis of Metal Element Distributions in Rice (*Oryza sativa* L.) Seeds and Relocation during Germination Based on X-Ray Fluorescence Imaging of Zn, Fe, K, Ca, and Mn

**DOI:** 10.1371/journal.pone.0057360

**Published:** 2013-02-22

**Authors:** Lingli Lu, Shengke Tian, Haibing Liao, Jie Zhang, Xiaoe Yang, John M. Labavitch, Wenrong Chen

**Affiliations:** 1 MOE Key Lab of Environmental Remediation and Ecosystem Health, College of Environmental and Resources Science, Zhejiang University, Hangzhou, China; 2 College of Chemistry and Life Science, Zhejiang Normal University, Jinhua, China; 3 Department of Plant Sciences, University of California Davis, Davis, California, United States of America; Kansas State University, United States of America

## Abstract

Knowledge of mineral localization within rice grains is important for understanding the role of different elements in seed development, as well as for facilitating biofortification of seed micronutrients in order to enhance seeds’ values in human diets. In this study, the concentrations of minerals in whole rice grains, hulls, brown rice, bran and polished rice were quantified by inductively coupled plasma mass spectroscopy. The *in vivo* mineral distribution patterns in rice grains and shifts in those distribution patterns during progressive stages of germination were analyzed by synchrotron X-ray microfluorescence. The results showed that half of the total Zn, two thirds of the total Fe, and most of the total K, Ca and Mn were removed by the milling process if the hull and bran were thoroughly polished. Concentrations of all elements were high in the embryo regions even though the local distributions within the embryo varied between elements. Mobilization of the minerals from specific seed locations during germination was also element-specific. High mobilization of K and Ca from grains to growing roots and leaf primordia was observed; the flux of Zn to these expanding tissues was somewhat less than that of K and Ca; the mobilization of Mn or Fe was relatively low, at least during the first few days of germination.

## Introduction

Micronutrient deficiencies, particularly those involving iron (Fe) and zinc (Zn), are the most prevalent deficiency-related health disorders in the world [1], [2]. Low micronutrient densities in staple foods are generally the major reasons for human micronutrient malnutrition in developing countries [3], [4], [5], [6], [7]. Therefore, biofortification of micronutrient contents in staple foods through agronomic or genetic approaches has attracted increasing interest during the past decades [3], [4], [7], [8]. Rice (*Oryza sativa* L.) is the most important food crop in the world, providing over 21% of the calory intake needs of the world’s population and up to 76% for the population of South East Asia [9]. Thus, the enhancement of rice grain micronutrient contents is viewed to be an important goal as part of global strategies for improving the nutritional quality of human diets.

The ability of cereals to supply dietary micronutrients largely depends on their concentrations and bioavailability in the grain. Distribution patterns of micronutrients within the grains are important because a considerable portion of the whole grain generally is removed and lost during processing (milling) before consumption [10] and mineral concentrations often decrease from the outer bran layers toward the endosperm [4]. For instance, in rice grains the mineral elements are highly concentrated in the outer layers [11], with rice bran containing 61% of the total grain mineral fraction [12]. However, most studies of mineral element compartmentation in rice grains have relied on bulk analysis, thus they do not provide detailed information on the distribution of the mineral elements in the different parts of the whole grain [8], [11], [12]. An *in vivo* imaging of mineral elements localization in rice grains should provide clearer indications of possible strategies controlling the bioavailability of important mineral elements (e.g., Zn and Fe) for human health.

Efficient mineral accumulation in seeds is essential for normal germination and establishment of the next vegetative rice plant generation [13]. For instance, Fe demand is high during the reproduction phase of the rice life cycle [14], and the nutrients stored in seed structures are mobilized during germination, suggesting that mineral elements are crucial for seed germination [15]. However, few reports described the details of the mineral element fluxes into the new, growing and differentiating seedling cells and tissues. Thus, a second aim of this study is to describe the germination-associated movements of individual mineral elements from their sites of accumulation in mature rice seeds into seedling plants.

However, the visualization of elements in plant tissues is not readily carried out; the concentrations of micronutrient elements often are quite low and the commonly used methods such as chemical imaging (e.g., [8], [16] generally lack sufficient sensitivity and/or resolution. Trace element analysis requires a technique that has detection limits as low as a few micrograms per gram. Synchrotron-based X-ray microfluorescence (μ-XRF) is a suitable tool for investigating the localization of mineral elements in cells and tissues due to its characteristics of high resolution and sensitivity [17], [18]. For example, μ-XRF has been used to investigate differential metal distribution patterns in wild-type and hyperaccumulating genotypes of *Sedum alfredii* [19], [20], [21]. Recently, spatial unloading of As within grains was also investigated by using SXRF microtomography [22]. In the present study, we used the sensitive µ-XRF technique for analysis of nutritionally relevant metals in different tissues of rice grains and the mobilization of these elements from seeds into developing rice seedling tissues in the early stages of germination.

## Materials and Methods

### Plant Materials

The seeds of rice IR68144, kindly supplied by Dr. Glenn Gregorio, the International Rice Research Institute (Manila, The Philippines), were used for this study. IR68144 is a high Zn and Fe density genotype, Fe and Zn in the unpolished rice grains of IR68144 was about 18 and 37 mg kg^−1^, respectively [23]. The seeds of IR68144 were recultivated in a greenhouse, southern China, with standard cultivation practice until harvest.

### Elemental Analysis

Rice grains were dehusked using a roller sheller (Model JLGJ4.5, Taizhou Grain Industry Instrument Corp, Zhejiang Province, China) and damaged kernels were removed with tweezers. A subset of the brown rice kernels was subsequently milled into polished rice and rice bran, the latter consists of aleurone layer plus embryo, using a JNMJ3 rice polisher (Taizhou Grain Industry Instrument Corp, Zhejiang Province, China). All the rice grains-derived samples (grains, brown rice, bran and polished rice) were milled into powder with a mixer mill (Retsch MM301, German) and oven-dried at 65°C for 72 h. Dried powders (0.1 g) were digested with 4.0 mL of HNO_3_ (reagent grade) and 1.0 mL of H_2_O_2_ (30%, analytical reagent, Beijing Chemical Works, China) in a screw cap polypropylene sample tube (Corning Incorporated, Corning, NY, USA) using a Hot Block Digestion System (Model SC154, Environmental Express, Mt. Pleasant, SC, USA). The duration of heat digestion is of 0.5 h at 80°C, and 2 h at 125°C. Each digest then was transferred to a 20 ml volumetric flask, made up to volume by water and filtered. The concentrations of elements in the samples (i.e., Ca, Mn, Fe, Zn, and K) were determined using inductively coupled plasma mass spectrometry (ICP-MS; Agilent 7500a, Agilent Technologies, Palo Alto, CA, USA), Standard Reference Materials 1568^a^ Rice Flour (National Institute of Staandards & Technology) containing Ca, Fe, K, Mg, Na, Mn, and Zn etc. was used, and the recovery of the elements was 90.2%∼108.4% of the certified value during the ICP-MS analysis. All materials were determined in four replicates.

### µ-XRF

#### Sample preparation

Seeds were grown on agar plates without nutrients, and harvested at 12 h or 48 h after sowing. The rice seeds with or without germination were cut with a cryotome (LEICA, CM1850) into about 200-µm sections at a temperature of −20°C. Briefly, grain samples were frozen using liquid nitrogen, and fixed immediately onto specimen disks using deionized water on a actively cooled (−40°C) specimen quick freezing shelf. The grains were first fractured with the blade longitudinally to obtain a flat surface, by which the grains were then refixed on the specimen disks for final sectioning. The two-step process is important for prevention of seed fracture and pulverization. Sections in good conditions were selected and freeze-dried (−20°C for 3 d).

#### μ-XRF analysis

Imaging of the distribution of elements (Zn, Fe, K, Ca, and Mn) was carried out using Synchrotron-based X-ray microfluorescence at Beamline 15 U (BL15U), Shanghai Synchrotron Radiation Facility (SSRF), China. The electron energy in the storage ring was 3.5 GeV with a current range from 200 to 300(5) mA. The brilliance of the beam in BL15U was 0.5×10^12^ photons/s/mm^2^/mrad^2^/0.1%BW. The microfocused beam of 3.5 µm was provided by a Kirkpatrick-Baez mirror pair (Xradia Inc.) with the sample at 45° to the incident X-ray beam. The fluorescence yield was detected using a 7-element Si (Li) solid state detector, positioned at 90° to the beam line. Dwell time per point was 0.5 s. The step size was set to 10 µm. Scanning areas of the samples were selected and observed using a microscope. BL15U saves full XRF spectrum data at each pixel. Elemental distributions are achieved by windowing on the elements of interest in the XRF spectra. The windows can be applied during data collection or during data analysis since the full XRF spectrum is saved for each pixel. The beam energy was set to 13 Kev during mapping. The fluorescence energies windowed for this investigation were Zn, Fe, K, Ca, and Mn. The fluorescence data are presented as color maps, and pixel brightness is displayed in RGB, with the brightest spots corresponding to the highest element fluorescence.

### Statistical Analysis

All data were statistically analyzed using SPSS (Version 12.0). The figures were made using the software Origin 8.0.

## Results

### Total Concentrations of Minerals in Different Fractions of Rice Grains

**Figure 1:**
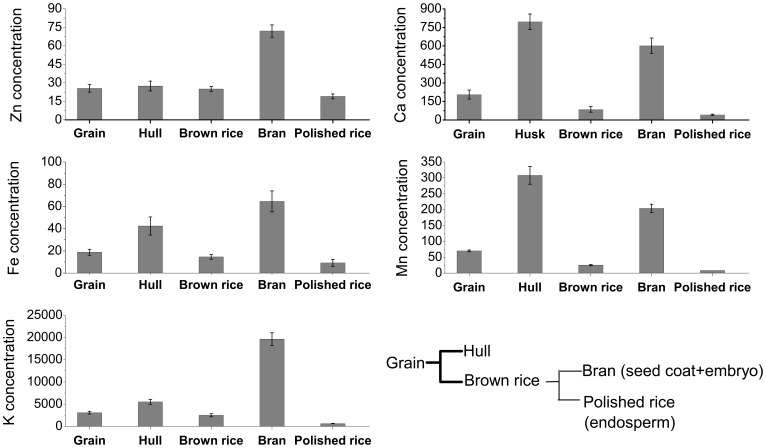
Elemental concentrations of rice grains, hull, brown rice, bran, and polished rice. All concentrations are expressed as mg kg^−1^ DW. Data points and error bars represent means and SEs of four replicates.

The concentrations of Zn, Fe, K, Ca and Mn were determined in different parts of rice grains after separation of whole grains into hulls and brown rice, and then milling the brown rice into bran and polished rice. The polished rice fraction is essentially the rice grain endosperm,and the bran contains most of the embryo and aleurone layer. As shown in Figure 1, the relative concentrations of Zn, Fe and K in the different grain fractions were: bran > hull > whole grain > brown rice > polished rice, whereas the relative concentrations of Mn and Ca were in the order: hull > bran > whole grain > brown rice > polished rice. The whole rice grain was essentially composed of hull, bran (embryo + aleurone layer) and polished rice (endosperm). The concentration of each of these elements in the polished rice (endosperm) was lower than that of the corresponding hull and bran (embryo + aleurone layer). The bran Zn concentration was 3 times the concentrations in the hull and polished rice. Variations in the other elemental concentrations in the different rice fractions were even higher than those for Zn. The Fe and K concentrations in the bran were 7 and 32 times their concentrations in polished rice, respectively. Likewise, the concentrations of Ca and Mn in the hull and bran were 14.3- to 38.5-fold higher than those in the polished rice.

**Figure 2:**
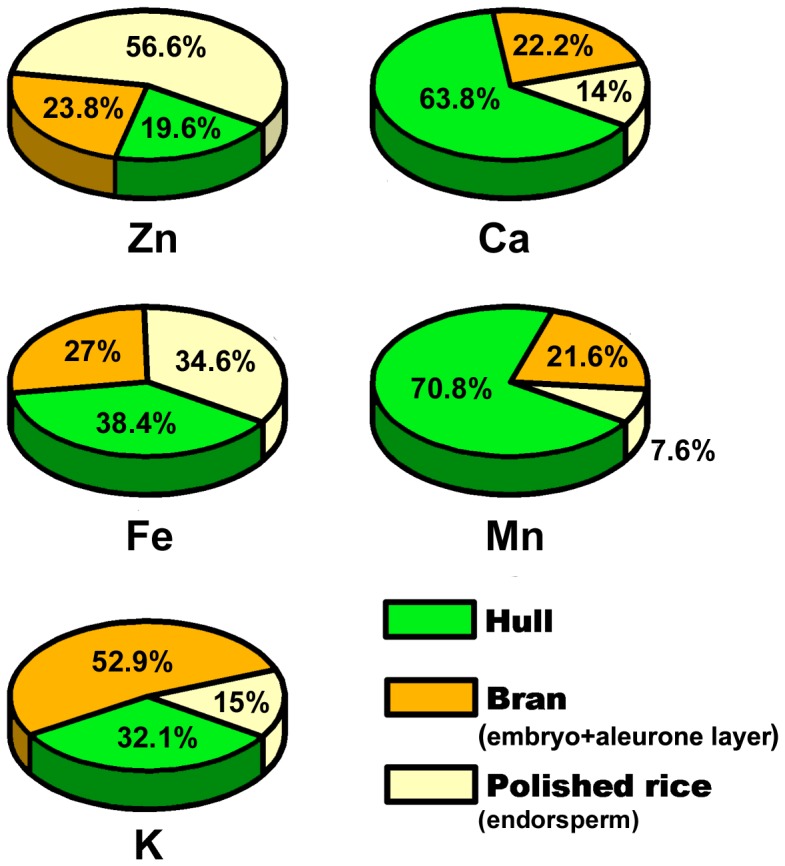
Percentage distributions of Zn, Fe, K, Ca, and Mn in different fractions of rice grains. Data are the means of four replicates.

The percentage distributions of the individual elements (Zn, Fe, K, Ca, and Mn) in the the three rice grain fractions were calculated according to the elemental concentrations and the corresponding weights of the fractions. The average weight percentage for hull, bran and polished rice was 17.9%, 8.3% and 73.8% of the total whole grain weight, respectively (data not shown) and the calculated percentage distributions are shown in Figure 2. More than half of the total Zn presented in the polished rice (endosperm), while Fe was relatively evenly distributed in the hull, bran and polished rice. The element K was preferentially distributed to the bran, followed by the hull, with least in the polished rice. Both Ca and Mn were mainly located in the hull, of 63.8% and 70.8%, respectively. Only 14% of the total Ca and 7% of the total Mn remained in the polished rice.

### In vivo Spatial Imaging of Minerals Distributions in Rice Grains

**Figure 3:**
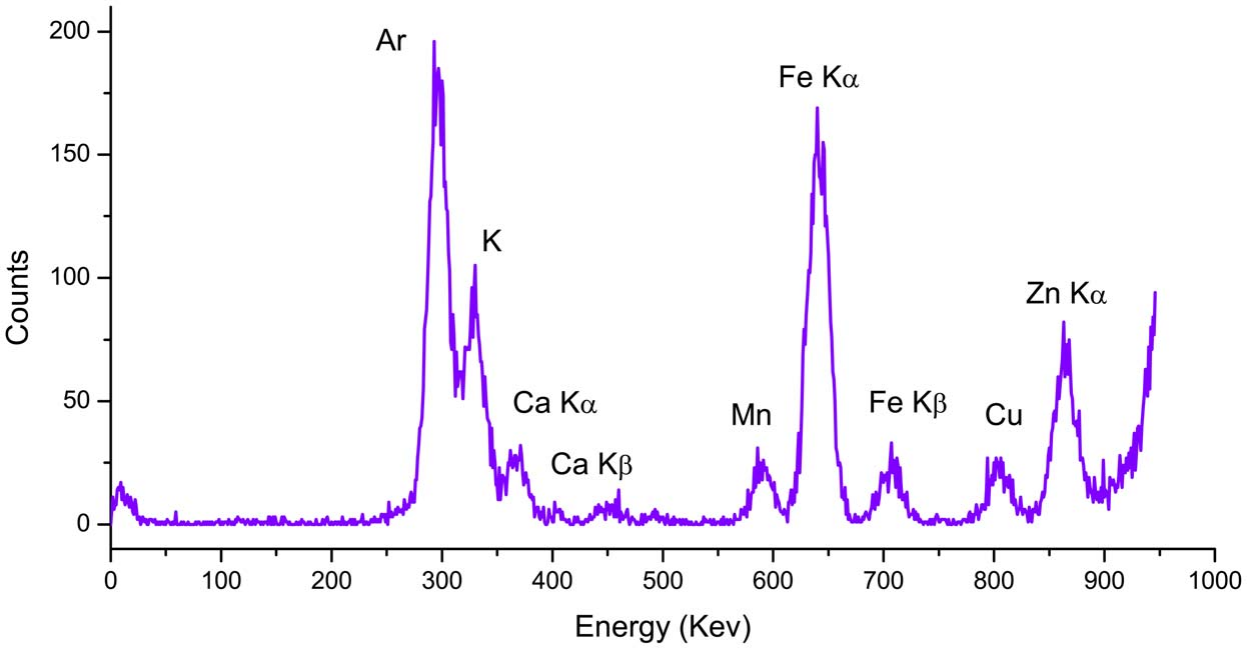
Typical synchrotron radiation X-ray fluorescence (SR-XRF) microprobe spectrum in an embryo localized scanning point of rice grain.

**Figure 4:**
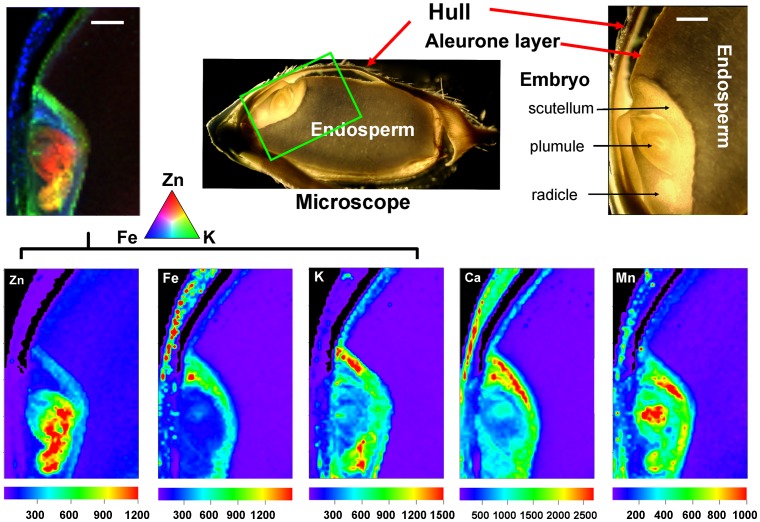
µ-XRF elemental maps for Zn, Fe, K, Ca, and Mn in rice grains. The green rectangle in the primary microscope image (top, center) has been rotated ca 75° counterclockwise to give the enlarged, labeled image (top right). This also is the orientation of the individual element distribution maps (lower row) and the color merged image (upper row, left). The fluorescence yield counts were normalized by I0 and the dwell time and maximum values vary for each element (scales beneath each false color map). Pixel brightness is displayed in RGB, with the brightest spots corresponding to the highest element fluorescence. The color-merged image shows the relative locations of Zn (red), K (green), and Fe (blue), as indicated by the colored triangle scale.

To investigate the distribution of mineral nutrients in rice grains, micro XRF mapping was performed. The X-ray fluorescence spectrum from a scanning point on the embryo of a rice grain showed clear peaks for K, Ca, Mn, Fe, Cu, and Zn (Fig. 3). The integrated intensity for each element was calculated from the spectrum and normalized by the intensity of the Compton scattering peak. Elemental mapping for the measurement area was obtained from the normalized intensity for each element. The elemental distribution maps of Zn, Fe, K, Ca, and Mn in the scanned areas of different regions of rice grains are presented in Figure 4, together with photographs taken using an optical microscope. Each map indicates the relative distribution of a specific element, and the scales of fluorescence counts vary for each map. The quantitation of the fluorescence yields was normalized by I0 and the dwell time. The normalized X-ray fluorescence intensities were scaled to different color brightness for individual elements, with the brightest spots corresponding to the highest elemental fluorescence.

As shown in Figure 4, the *in vivo* scanning maps for Zn, Fe, K, Ca, and Mn in the longitudinal section of the rice grain are consistent with the quantitative elemental distribution results from the ICP-MS analysis. Although the general accumulation of the different mineral elements was low in the endosperm, the distribution patterns varied considerably in the other parts of the rice grain. Concentrations of all the elements were high in the embryo regions even though the local distributions within the different parts of the embryo varied. Zinc was mainly found in the central parts of the embryo (the plumule and radicle), whereas the highest intensity of Fe was observed in the scutellum of embryo and hull, followed by the aleurone layer and the other parts of the embryo. Potassium was highly concentrated in the embryo, particularly in the scutellum. The merged color image for Zn (red), Fe (blue) and K (green) clearly shows the significant differences in their relative distribution patterns within the embryo. Calcium was preferentially distributed to the hull and scutellum, while Mn was mainly localized to the hull and the embryo, the latter including its radicle, plumule and scutellum.

### Mobilization of Seed-accumulated Metals during Germination

**Figure 5:**
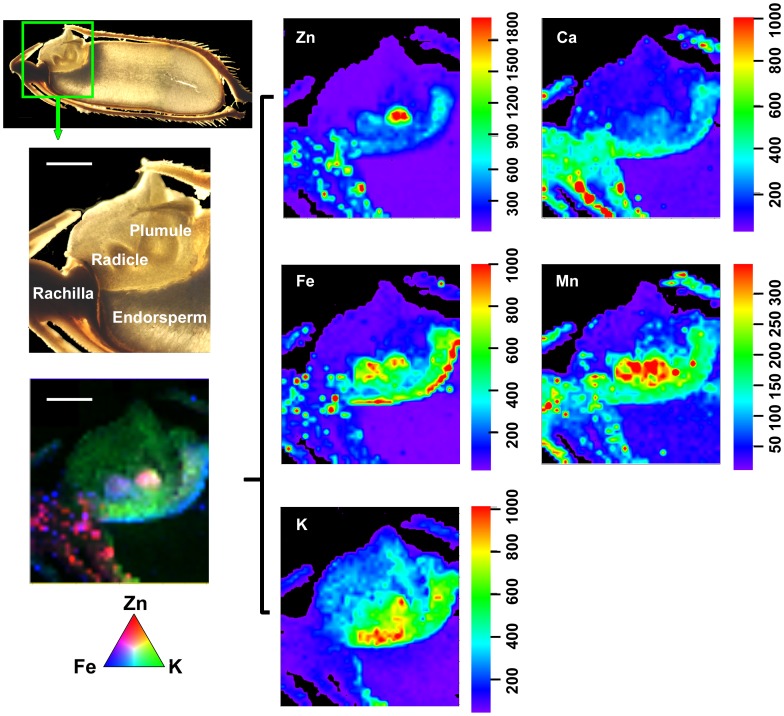
µ-XRF elemental maps of rice grains after germination for 12 h. The green box on the lower magnification photo of the seed (upper, left) was rotated 90° clockwise to give the labeled, higher magnification image as well as the individual µ-XRF elemental maps and the color-merged image (lower left). Refer to the legend for Figure 4 for additional details.

**Figure 6:**
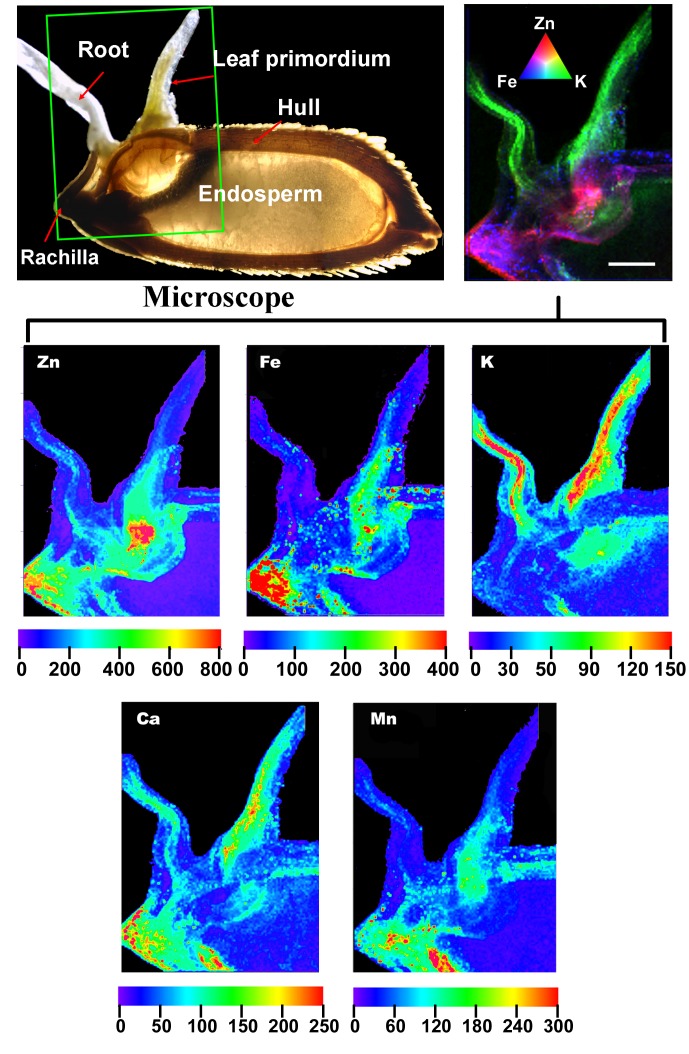
µ-XRF elemental maps of early stage rice seedlings after seed germination for 48 **h.** The orientation of the individual µ-XRF elemental maps and the color-merged image (upper right) is the same as that of the green rectangle around a portion of the labeled image of the germinating seed. Refer to the legend for Figure 4 for additional details.

Changes in the seed distribution patterns of each element at 12 h and 48 h after germination were determined. The changes in element distribution differed for the different elements monitored (Figs. 5 and 6). For Zn, Fe, Ca and Mn, the highest concentrations were observed in the rachilla, the short, sterile stem-like structure that bore the spikelets and now remains as part of the hull. However, the greatest concentration of K was in the radicle and plumule regions of the embryo, a localization that is most obvious at 48 h post germination (Fig. 6) when the root and shoot have begun to elongate. Mobilization of Zn, Fe, Ca and Mn to the elongating seedling organs was relatively low at 48 h. While there appeared to be relatively little accumulation of Fe, Zn and Mn in the elongating radical, these elements did accumulate in the elongating shoot tissues. However, there was a clear mobilization of Ca to the radicle, although the movement of Ca into the elongating shoot tissues was greater.

## Discussion

The rice grain consists of four major tissues: the hull, embryo, aleurone layer, and starchy endosperm. The endosperm is the most important grain fraction with respect to human nutrition, as it is the part of the grain primarily consumed in many countries. However, the bulk analysis of mineral element contents in different sections of rice grains (Fig. 1), which agrees well with the more tissue location-specific µ-XRF mapping (Fig. 4), shows very low levels of mineral elements in the endosperm, with considerably more Zn, Fe, K, Ca, and Mn in the embryo, aleurone layer and hull tissues. Taking weight percentage into account, the data for the present study show that 43% of the total Zn, 65% of the total Fe, and 85%∼92% of the total K, Ca and Mn were removed by the milling process if the hull and bran tissues (embryo + aleurone layer) were thoroughly removed from the endosperm fraction during polishing (Fig. 2). This is consistent with the previous study on distribution of mineral elements in three different rice cultivars [11]. Thus the removal of husk, embryo and aleurone layer during dehusking and polishing substantially reduces the mineral nutrient value of rice grains. Rice bran has been recognized as an extremely valuable source for nutrients due to its high contents of lipid, protein, vitamin, minerals, and dietary fiber [24]. The present study, therefore, supports the idea that brown rice or germinated brown rice is preferable from a health perspective because it contains more nutritional mineral components than polished rice [25]. It is recently reported that germinated brown rice has become one of the most interesting rice products and gained great attention, particularly in Asian countries, for its high nutritional value [26]. However, cautions should be taken for brown rice or rice bran consumption since rice bran may also be rich in toxic elements such as arsenic [22]. If the traditional practice of eating polished rice is to be continued, our results indicate that enhancement of micronutrient import into the endosperm should have a positive impact on the “mineral nutrient health” of human populations that obtain a lot of their daily calories from cooked rice. If that is so, our data further suggest that the continuing examination of the mechanisms that influence the transfer of mineral elements into the developing maternal tissues and their subsequent accumulation would be a much more important goal than simply increasing the mineral content of whole mature, harvested rice grains. At this time, however, knowledge of the mechanisms involved in mobilization of minerals into the different seed tissues during grain filling is limited.

The filial tissues (aleurone, endosperm, and embryo) of the rice caryopsis are generally isolated from the maternal tissues (e.g., rachilla, hull) by the absence of symplastic continuity [14] or direct vascular connections. For minerals to enter the filial tissues, several transport steps are required. These are, at a minimum: (1) phloem unloading into maternal tissues (i.e., ovule integuments that will develop into the seed coats, and nucellus), (2) efflux from the maternal seed coat cells into the apoplast surrounding the developing fertilized embryo sac, and (3) uptake into the filial tissues [14]. The results from the bulk analysis and μ-XRF mapping in this study showed that mineral nutrients were preferentially localized in the hull (Figs. 1, 4) and rachilla (Fig. 6) except for K and Zn. These observations support the conclusion that the maternal seed tissues are the first mineral storage sites during grain fill. Filling of the seeds is reported to occur through unloading from the phloem into the nucellar tissue followed by uploading into the aleurone layer, and transport into the endosperm [27]. The region of the ovular vascular trace presents on the ventral side of the ovary, which contains both phloem and xylem cells, is the point of entry into the filial tissue (the aleurone and the endosperm) for minerals [22], [27]. Elemental distributions in the outer bran layers are generally not uniform, but largely concentrated in the ovular vascular trace as reported previously [22]. This could also be observed from the images shown in the present study (Figs. 5 and 6). Dynamic changes in the distribution of minerals during rice seed development has been reported recently, but the role of OVT has not been determined [28]. Further studies of the transfer of nutrients into the filial tissues are necessarily involve imaging the ovular vascular trace by using transverse sections of grains. Inside the filial tissues of rice grains, our results showed that the minerals were preferentially localized to the embryo and/or aleurone layer (Fig. 4), resulting in higher concentrations of mineral elements present in the bran fraction compared with the polished rice (Fig. 2). The aleurone, which contains phytic acid rich granules, is thought to be a storage reservoir of positively charged metals such as Ca, Mg, K, Zn, and Fe, presumably for support of processes essential for seed germination and growth [29]. It has been reported that metals accumulate preferentially in the protein-rich aleurone and embryo tissues [30]. The embryo, the next generation, is the most important structure in seeds. Thus, from the perspective of the seed’s energy economy, it is probably more efficient to store mineral elements needed for enzyme functions, harvesting of light energy, maintenance of turgor as cells grow etc. in the embryo than to use energy for moving these elements from storage locations in the endosperm. Storage of these elements in the endosperm might also expedite the initiation of germination once the appropriate environmental conditions are detected. The µ-XRF mapping data (Fig. 4) support the conclusion that the key mineral nutrients are embryo tissue-localized.

While the embryo represents a high-density site for minerals accumulation, these mineral elements had largely non-overlapping regions of localization within the various regions of the embryo (Fig. 4). In the rice grain embryo, Zn was largely restricted to the plumule and radicle, whereas K, Fe, and Mn were preferentially localized to the scutellum. Distinct locations of elements such as Fe, Mn, and Zn have been observed in embryos of barley grains [10] and Arabidopsis seeds [31], with Fe in provascular strands, Mn on the abaxial sides of the cotyledons, and Zn distributed broadly throughout the embryo. Recently, a confocal 3D µ-XRF spectrometer was applied to rice grains, providing elemental maps for K, Ca, and Fe at different depths in the embryo, these maps indicated different element concentrations at different depths [32]. These distinct distributions of mineral elements possibly result from their various usages during seed germination and growth.

In the present study, we examined changes in the distributions of Zn, Fe, K, Ca, and Mn after the initiation of rice seed germination (Figs. 5 and 6), presumably suggesting locations in the embryo tissues where the elements had germination-specific roles to play [33]. During germination the observed shifting patterns of mineral distribution suggested different cellular capacities for element mobilization, as follows: K>Ca>Zn>Mn>Fe (Figs. 5, 6). High concentrations of K and Ca in the newly developed roots and leaf primordium during seed germination were observed (Fig. 6). Both K and Ca are essential plant macronutrients and are of high requirement for proper growth and reproduction of plants. Potassium has an important role in the activation of many growth related enzymes, may be important for allowing growing cells to maintain a turgor pressure in excess of the cell wall yield threshold, and is generally of high mobility in plants. Higher viability and vigor of seeds soaked in K solution has been reported [34]. It is thus not surprising that high remobilization of K occurs during rice seed germination to assure growth of the soil and light-seeking axes. Rapid movement of Ca to the new growing tissues during rice germination, however, contrasts with the general understanding that Ca mobility is relatively limited in plants. Calcium is an important element involved in cell wall structure and cell growth, as well as a central regulator of plant cell signaling [35], serving as an important second messenger involved in signal transduction of many phytohormones, including abscisic acid, which regulates many aspects of plant development including seed germination and seedling growth [36]. The high remobilization of Ca during rice seed germination stage that is inferred from the data in Figures 5 and 6, suggests a large requirement of Ca for efficient uses in the highly metabolically active differentiating cells. Zinc is also highly mobile during rice germination, and may be utilized in protein synthesis and functions, membrane structure and functions, gene expression and oxidative stress tolerance in the new growing tissues [16], [37]. Preferential distribution of Zn in the central parts of plumule and radicle suggests a critical role of Zn in either the meristemmatic and/or vascular tissue, which occupy the central axis of these structures. During seed germination, the transport of Zn may be required for a high level of meristemmatic activities. Relatively low remobilization of Fe and Mn were observed in rice grains during germination (Fig. 6). One of possible reasons for the low level of Fe redistribution may be the vacuolar sequestration of the element. Free Fe in the cell prompts the formation of excess reactive oxygen species, which cause damage to subcellular organelles. Fe is stored in vacuoles until it is needed, close to the site of assimilation. The vacuolar storage of Fe in Arabidopsis seeds, mediated by a vasuolar membrane transporter VIT1, is critical for seedling development [31]. Recent studies also showed that functional disruption of vacuolar membrane transporters OsVIT1 and OsVIT2, which highly expressed in flag leaf blade and sheath, leads to increased Fe accumulation in rice seeds [38]. During germination, Fe is required for respiration, and its release from storage vacuoles may occur as a result of a genetic signal that causes upregulation of Fe export proteins such as NRAMP3, which located on the vacuolar membrane to move the metal out of the vacuole for use [39].

Knowledge on these preferential elemental constitutions of the different grain tissues and their remobilization patterns during germination makes the possibility of designing target products with nutritionally optimal constitution more feasible. The result may also be useful in order to optimize various germination procedures (both processes and management strategies) of edible seeds, in order to enhance the nutritional quality of food mixtures based on coarse cereals [40], [41]. However, at present much more work is needed for a better understanding of the important roles of minerals involved in the rice seed development and seed germination.
